# Microbial detoxification of mycotoxins in food

**DOI:** 10.3389/fmicb.2022.957148

**Published:** 2022-11-23

**Authors:** Nadine Abraham, Edicon Tze Shun Chan, Ting Zhou, Stephen Y. K. Seah

**Affiliations:** ^1^Department of Molecular and Cellular Biology, University of Guelph, Guelph, ON, Canada; ^2^Guelph Research and Development Centre, Agriculture and Agri-Food Canada, Guelph, ON, Canada

**Keywords:** Mycotoxins, aflatoxins, ochratoxin, citrinin, zearaleneone, patulin, deoxynivalenol, T2

## Abstract

Mycotoxins are toxic secondary metabolites produced by certain genera of fungi including but not limited to *Fusarium*, *Aspergillus,* and *Penicillium*. Their persistence in agricultural commodities poses a significant food safety issue owing to their carcinogenic, teratogenic, and immunosuppressive effects. Due to their inherent stability, mycotoxin levels in contaminated food often exceed the prescribed regulatory thresholds posing a risk to both humans and livestock. Although physical and chemical methods have been applied to remove mycotoxins, these approaches may reduce the nutrient quality and organoleptic properties of food. Microbial transformation of mycotoxins is a promising alternative for mycotoxin detoxification as it is more specific and environmentally friendly compared to physical/chemical methods. Here we review the biological detoxification of the major mycotoxins with a focus on microbial enzymes.

## Introduction

Mycotoxins are secondary metabolites produced by several genera of filamentous fungi and are toxic to animals at low concentrations (Stoev, 2013). These toxins producing fungi typically infect crops, including cereals, nuts, oilseeds, and fruits (Table 1; Stoev, 2013). Since many of these crops are also used to feed farm animals, meat and animal products including milk and eggs can also become contaminated by mycotoxins (Zain, 2011). Ingestion of mycotoxin contaminated food poses a health safety risk for humans and livestock, causing mycotoxicosis. This can result in death and chronic effects that may lead to cancer or developmental defects. Besides their negative impact on human and animal health, mycotoxins are an economic burden to the agriculture industry due to reduced crop productivity and value, as well as the cost of regulatory programs to ensure that these toxins are not introduced into the food chain (Zain, 2011). Indeed, many countries have implemented legislation and guidelines to limit the levels of mycotoxins found in food and feed commodities. Nevertheless, it is estimated that mycotoxins can be detected in 60%–80% of food grains and up to 20% could be above regulatory limits (Eskola et al., 2020).

**Table 1:** Common mycotoxins that contaminate food and the fungi that produce them.

Mycotoxins	Fungal producers	Main occurrence in food	References
Aflatoxins	*Aspergillus* species	Oilseed crops including corn, peanuts, tree nuts, and cottonseed. Can accumulate in meat and animal products due to consumption of contaminated grains	Mahato et al. (2019)
Zearalenone	*Fusarium* species	Grain cereals like maize and wheat	Ji et al. (2019)
Citrinin	*Aspergillus*, *Penicillium*, and *Monascus* species	Grains such as rice, maize, and wheat	Silva et al. (2021)
Patulin	*Penicillium*, *Aspergillus* and *Byssochlamys* spp.	Pomme fruit, fruit products, and cheeses	Saleh and Goktepe (2019)
Ochratoxins	*Aspergillus ochraceus*, *Aspergillus ostianus*, and *Penicillium verricosum*.	Cereals, coffee, nuts, and grapes	Duarte et al. (2010)
Fumonisins	*Fusarium verticillioides* and *Fusarium proliferatum.*	Corn or corn products	Braun and Wink (2018)
Trichothecenes	*Fusarium*, *Trichoderma*, *Trichothecium*, *Stachybotrys*, *Myrothecium*, and *Spicellum* species	Cereals such as wheat, barley, and corn	Ji et al. (2019)

**Figure 1:**
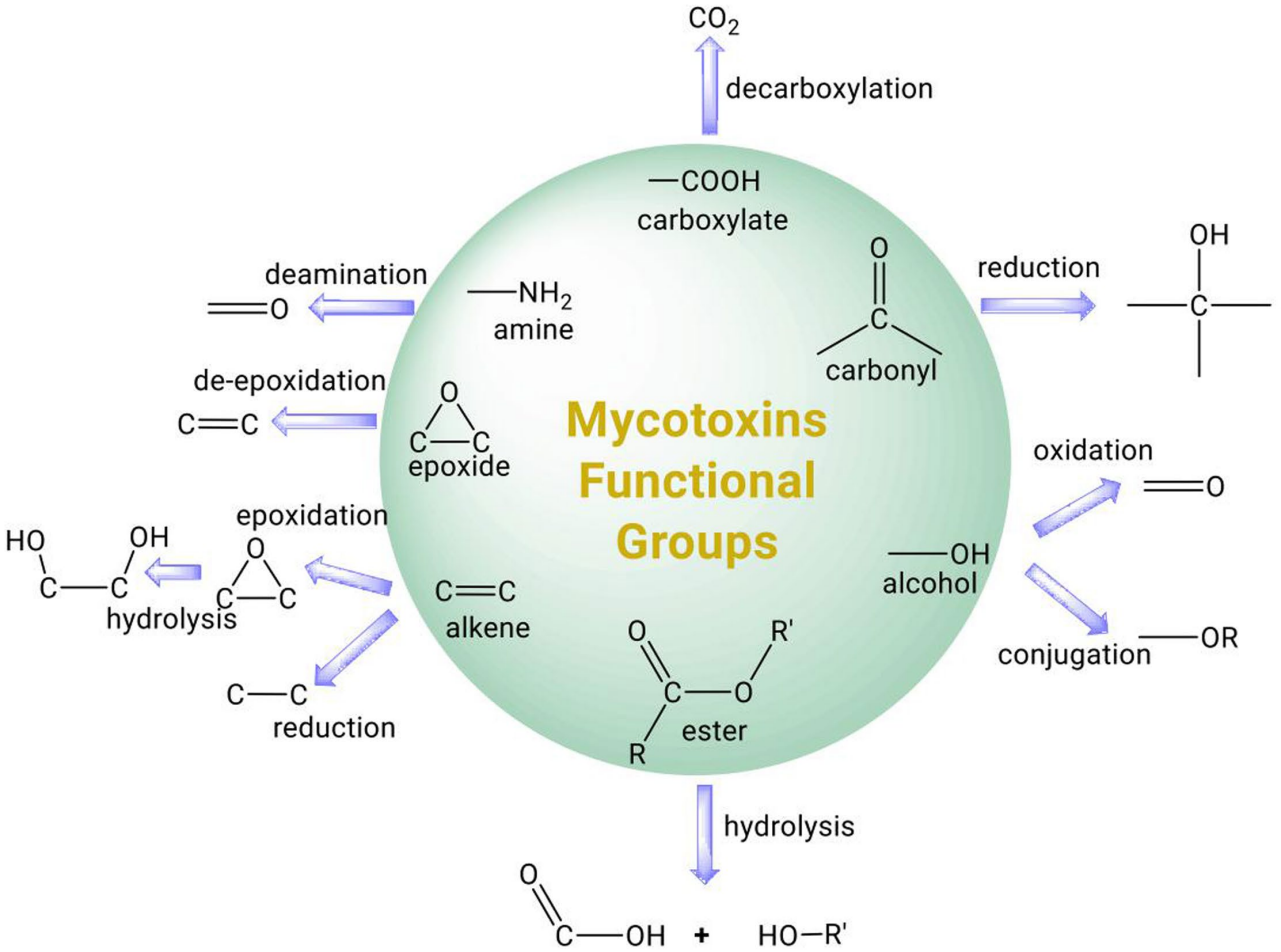
Summary of common functional groups of mycotoxins (circle) and their possible transformation by microbial enzymes.

Strategies to mitigate mycotoxin contamination include the use of fungi-resistant crops and the application of pesticides, fungicides, or biological control methods to limit the growth and transmission of fungal pathogens (Cleveland et al., 2003). However, mycotoxin contamination frequently occurs post-harvest as the environmental conditions for the storage of agri-food commodities, such as grains, are often conducive to the growth of molds (Neme and Mohammed, 2017). Chemicals such as ammonia and ozone can react with mycotoxins to form less toxic products, but they leave chemical residues and can have negative effects on nutritional quality (Chelkowski et al., 1981; Conte et al., 2020). Physical methods for mycotoxin removal involve the use of washing, heat, radiation, or adsorbents, such as clay, activated carbon, or microbial cells (Stoev, 2013). In particular, the use of adsorbents that can bind to mycotoxins without dissociating in the digestive tract has been applied successfully in animal feed (Vila-Donat et al., 2018). Prophylactic use of calcium montmorillonite clay has also shown some promise in human clinical trials and may be useful for individuals at high risk of developing aflatoxicoses from consumption of contaminated food (Afriyie-Gyawu et al., 2008; Wang et al., 2008; Phillips et al., 2019). Several recent reviews on the use of microbial cells as adsorbents of mycotoxins are available (Vila-Donat et al., 2018; Xu et al., 2022) and therefore this topic is not covered in this review. Due to their rapid evolution and metabolic plasticity, microorganisms have the capability to degrade a large number of organic compounds and xenobiotics. Not surprisingly, both bacteria and fungi have been isolated from soil, the digestive tract of animals, or mycotoxin-contaminated products that are capable of transforming mycotoxins (Zhu et al., 2017). The toxicity of mycotoxins is often attributed to specific functional groups in their chemical structure. Therefore, selective modification of these functional groups by microbial enzymes could reduce or eliminate the mycotoxins toxicity (Figure 1). Microorganisms and their associated enzymes are therefore potentially useful for mycotoxin detoxification in food as they are specific and capable of catalyzing the biotransformation of mycotoxins under benign environmental conditions. Here, we provide a comprehensive review of the transformation of major mycotoxins by microbial enzymes and discuss their potential application for mycotoxin detoxification in food and feed. Many of the microbial enzymes involved in mycotoxin transformation require cofactors or co-substrates and we address their significance for the successful implementation of enzymatic strategies to remove mycotoxins in food/feed.

## Aflatoxins

**Figure 2:**
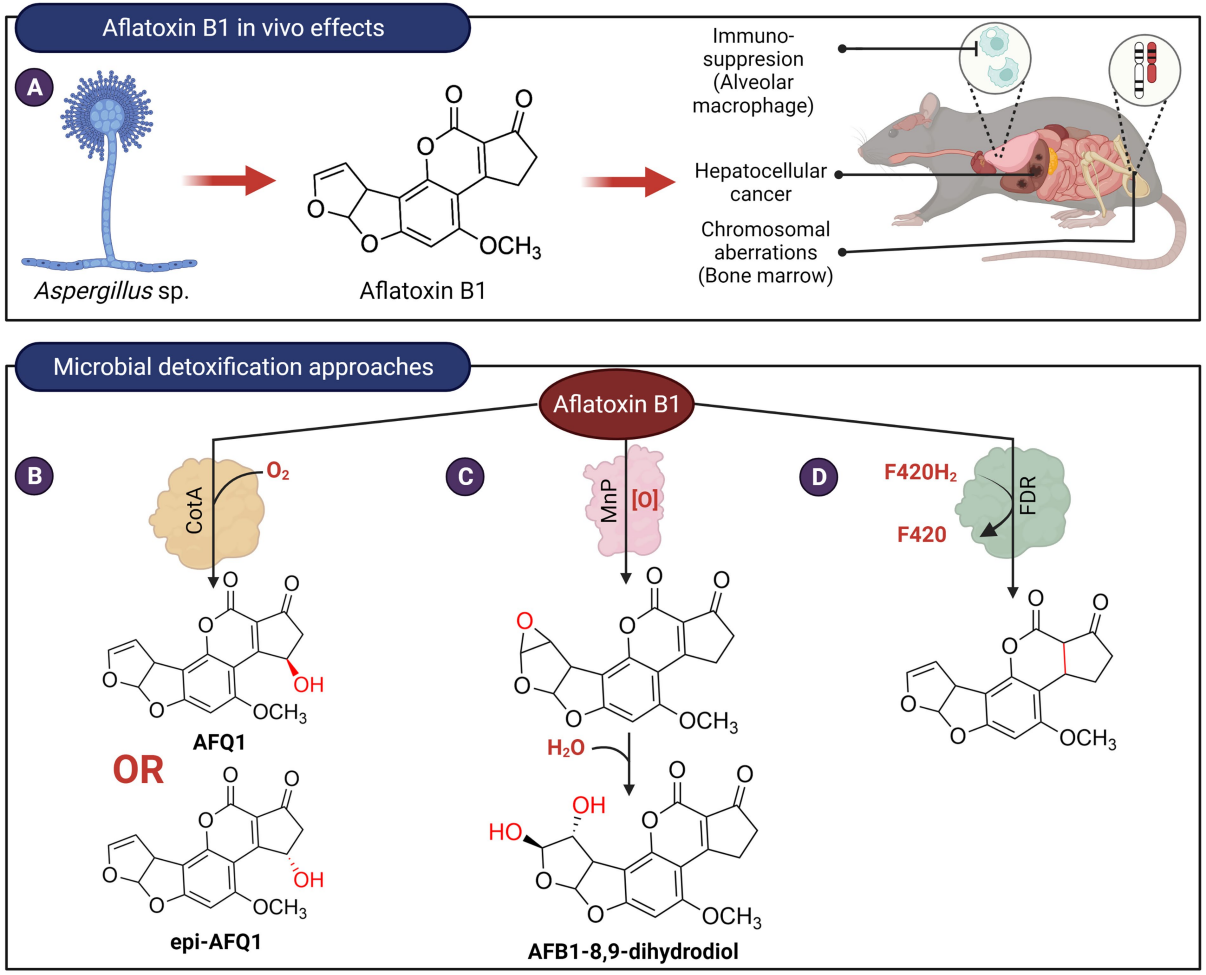
*In vivo* effects of aflatoxin B1 and microbial detoxification approaches. **(A)** Aflatoxin B1, produced by members of *Aspergillus* is carcinogenic, genotoxic, and possesses immunosuppressive properties. **(B)** *Bacillus licheniformis* ANSB821 utilizes the laccase, CotA to biotransform aflatoxin B1. **(C)** The manganese peroxidase MnP from the white-rot fungus *Phanerochaete sordida* YK-624 oxidizes AFB1 to form AFB1-8,9-dihydrodiol *via* an 8,9-epoxide intermediate. **(D)** Proposed reduction of AFB1 ring by a deazaflavin cofactor (F_420_) containing *M. smegmatis* enzyme.

Aflatoxins are difuranocoumarin mycotoxins that are produced by various species of *Aspergillus* that frequently associate with crops such as oilseed, corn, peanuts, etc. under warm and humid conditions (Mahato et al., 2019). Due to their fat solubility, these toxins can accumulate in animals exposed to contaminated feed, resulting in their occurrence in meat and other animal products (Pickova et al., 2021). Exposure to aflatoxins in animals can cause acute aflatoxicosis and death, while long-time exposure to low concentrations may cause cancer or lower performance and production of farm animals. The main aflatoxins of significance are aflatoxins B1, B2, G1, and G2, with aflatoxin B1 being the most toxic and the most potent liver carcinogen. AFB1 can be transformed by microsomal cytochrome P450 enzyme, inducing cellular oxidative stress and forming highly reactive AFB1-exo 8,9-epoxide that can form adducts with DNA and other cellular macromolecules, thereby contributing to its carcinogenic, teratogenic and mutagenic activity (Eaton and Gallagher, 1994; Figure 2A). In the liver, AFB1 and AFB2 are also metabolized by microsomal monooxygenases to the hydroxylated derivatives AFM1 and AFM2. AFM1 and AFM2 can however be secreted in milk (Prandini et al., 2009). Although less carcinogenic than their parent compounds when tested on rats (Cullen et al., 1987), the hydroxylated derivatives of the aflatoxins have similar toxicity as their parent compounds when tested on rats (Pong and Wogan, 1971) and ducks (Purchase, 1967).

Enzymes that depolymerize polyaromatic lignin, such as laccases and peroxidases, have been shown to oxidize aflatoxins. These enzymes frequently require additional co-substrates or mediators that may limit their practical use for the decontamination of aflatoxins in food. These redox mediators are the true substrate of laccases, and when oxidized, they form radicals that can in turn react with aflatoxins. For example, studies showed that laccase from *Pleurotus eryngii* was not capable of oxidizing AFB1 in the absence of redox mediators (Loi et al., 2016, 2018). *Pleurotus pulmonarius* laccase can transform 23% of AFB1 but the addition of 1 mM redox mediators, 2,2′-azino-bis-3-ethylbenzothiazoline-6-sulfonic acid, or acetosyringone resulted in an increased transformation of AFB1 to about 45% (Loi et al., 2016). The involvement of redox mediators in the laccase-catalyzed reactions indicates that they are not specific to aflatoxins and may potentially react with other molecules found in food. Recently, a bacterial laccase, CotA, from *Bacillus licheniformis* ANSB821 was found to transform AFB1 into two 3-hydroxy epimers of AFB1, AFQ1 and epi-AFQ1, without any redox mediators (Guo et al., 2020; Figure 2B). These two epimers do not exert any toxic effects on human hepatic (L-02) cells. AFQ1 toxicity was also shown to be about 1% of AFB1 in rainbow trout (Eaton and Gallagher, 1994) and 18-times less toxic than AFB1 in chicken embryo assay (Hsieh et al., 1974). However, the mechanism for direct oxidation of a non-phenolic moiety of AFB1 by CotA remains paradoxical and requires further investigation.

Treatment of AFB1 with manganese peroxidase MnP from the white-rot fungus *Phanerochaete sordida* YK-624 resulted in the formation of AFB1-8,9-dihydrodiol *via* an 8,9-epoxide intermediate (Wang et al., 2011; Figure 2C). The mutagenicity of AFB1 following treatment by this enzyme was reduced by about 50%–70%, suggesting that the highly reactive and toxic 8,9-epoxide did not accumulate in the reaction. Like laccases, the transformation of AFB1 by MnP is thought to be indirect. It was speculated that MnP produced formate or superoxide anion radicals that in turn react with AFB1. Transformation of AFB1 is also enhanced by the presence of Tween 80 and not Tween 20 (Bao et al., 1994). The former contains unsaturated fatty acids, suggesting that AFB1 oxidation by MnP can also be mediated by lipid peroxyl radicals. A recombinant N246A variant of the dye-decolorizing peroxidase, DypB (Rh_DypB) from *Rhodococcus jostii* (Vignali et al., 2018) was also able to transform AFB1. LC–MS–MS results suggested that it oxidizes ring A of AFB1 to form a product likely to be AFQ1 (Loi et al., 2020). However, DypB requires hydrogen peroxide as a co-substrate and may be undesirable for use in food.

An oxidase from the mushroom *Armillariella tabescens* was previously reported to oxidize AFB1 (Liu et al., 2001). This enzyme is not homologous to known aflatoxin oxidases, such as laccases or peroxidases. Instead, it shares sequence and structural homology with the hydrolase, dipeptidyl dipeptidase III (Xu et al., 2017). Subsequent investigation revealed that this enzyme had peptidase activity and lacked the previously ascribed aflatoxin oxidative activity (Karačić et al., 2017).

The lactone moieties of AFB1 are thought to be important for its toxicity as cleavage of the lactone ring in AFB1 resulted in the loss of fluorescence and its mutagenicity and toxicity are reduced by 450- and 18-times, respectively (Lee et al., 1981). Eleven *Bacillus* species from pond mud and soil samples were able to reduce the fluorescence of AFB1 (González Pereyra et al., 2019). Three of the strains, *B. subtilis* RC1B, *B. cereus* RC1C, and *B. mojavensis* RC3B contained genes that are homologous to *aiiA*, a lactonase that can hydrolyze the lactone ring of the Gram-negative bacteria quorum sensing autoinducer, acyl-homoserine lactone. However, hydrolysis of the AFB1 lactone ring has not been demonstrated with purified AiiA. In addition, not all the *Bacillus* strains tested contained the *aiiA* gene, suggesting that other enzymes may be responsible for the observed hydrolysis of AFB1.

Lastly, nine *F*(420)-dependent reductases (FDR) from *Mycobacterium smegmatis* that utilize the deazaflavin cofactor F_420_H_2_ were found to catalyze the transformation of AFG1, AFG2, AFB1, and AFB2, with AFG1 being the best substrate for all nine enzymes (Taylor et al., 2010). The products as determined from LC–MS have an increased m/z of 2.02 indicative of the reduction of aflatoxin by 2 electron transfers from the F_420_H_2_ cofactor of the enzymes. It is suggested that the α,β unsaturated ring A of the aflatoxins was reduced by the enzyme (Figure 2D). However, the toxicity of the ring A saturated products in comparison to AFB1 has not been determined. In addition, this enzyme will require reducing agents to recycle the oxidized deazaflavin cofactor, which may not be practical for the decontamination of aflatoxins in food products.

## Zearalenone

**Figure 3:**
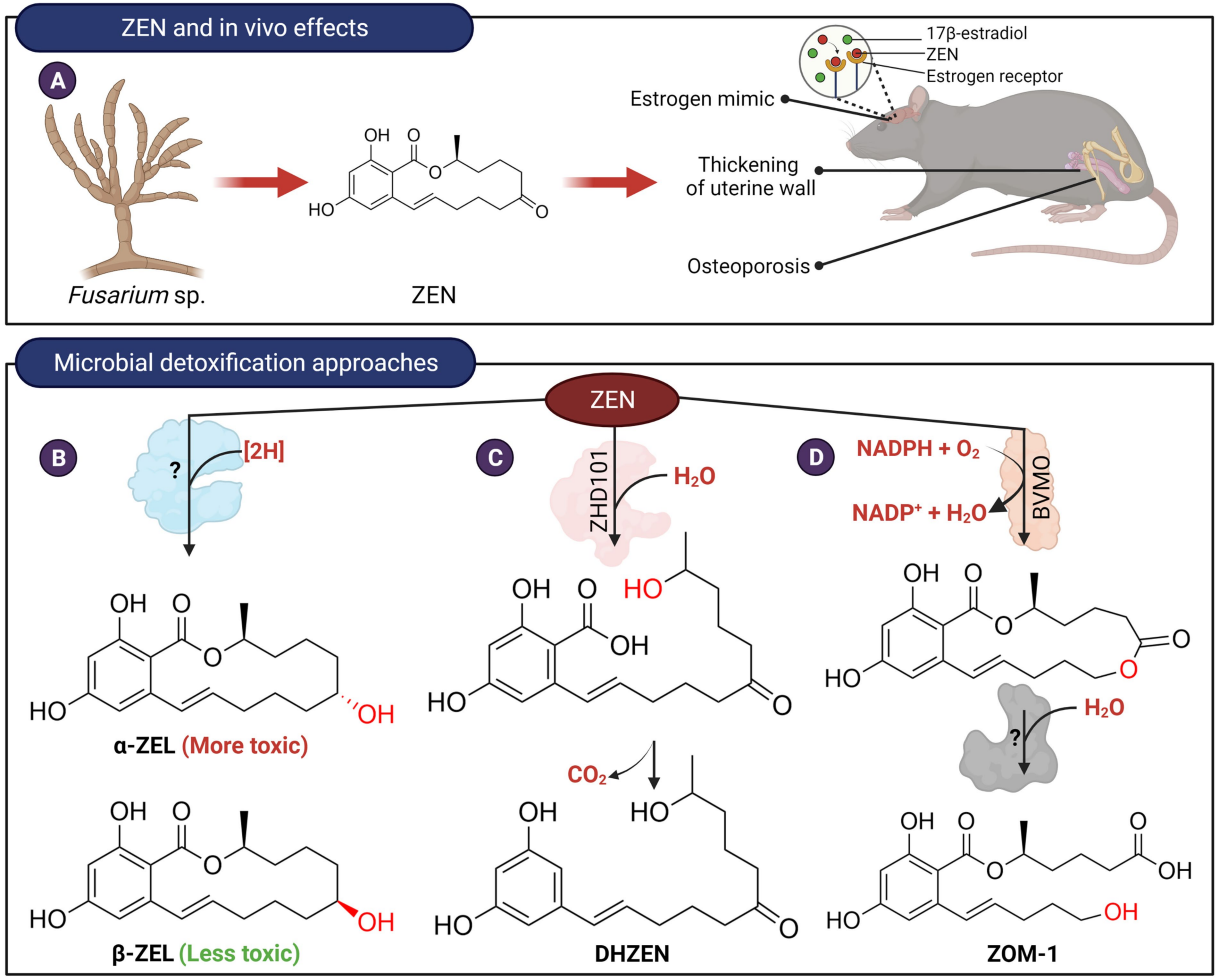
*In vivo* effects of ZEN and microbial detoxification approaches. **(A)** ZEN possesses estrogenic properties which in turn affect the endocrine and reproductive systems of animals. **(B)** Transformation of ZEN to α-ZEL or β-ZEL by microorganisms. The C7-carbonyl oxygen is reduced to an alcohol stereospecifically (red). The enzyme(s) responsible for catalyzing this reaction have not been isolated and identified. **(C)** ZEN can be hydrolyzed by lactone hydrolases followed by spontaneous decarboxylation to produce DHZEN. **(D)** Transformation of ZEN to ZOM-1 was postulated to occur in two steps. The first step could potentially be catalyzed by a Baeyer-Villiger type monooxygenase. The lactone product can then be hydrolyzed to ZOM-1.

Zearalenone (ZEN) is a α,β-resorcylic acid lactone, and its toxicity is attributed to its estrogenic properties (Metzler et al., 2010; Figure 3A). Various microorganisms, including gut microbes, were able to reduce the C6 keto group of zearalenone to produce α-zearalenol (α-ZEL) and β-zearalenol (β-ZEL; Figure 3B; El-Sharkawy and Abul-Hajj, 1988; El-Sharkawy et al., 1991). While β-ZEL is less toxic than ZEN, α-ZEL binds to estrogen receptors 10–20 times stronger and exhibits >90-fold higher estrogenic activity, compared to ZEN (Metzler et al., 2010). The enzyme(s) responsible for the reduction of ZEN has not been identified and therefore it is not clear if the two activities can be separated.

Detoxification of ZEN can also occur through modification of the C14-hydroxyl group or hydrolysis of the lactone ring as these moieties are important for the estrogenic property of ZEN (El-Sharkawy and Abul-Hajj, 1988). For example, ZEN can be conjugated with glucoside and sulfate at the C14-hydroxyl groups by plants and various microbial species (Kamimura, 1986; El-sharkawy and Abul-hajj, 1987; El-Sharkawy et al., 1991; Plasencia and Mirocha, 1991; Jard et al., 2010; Brodehl et al., 2014; Paris et al., 2014). Although these esters are not estrogenic, they can be hydrolyzed to the parent compound by gut microbiota and are therefore not considered a viable method to reduce the toxicity of ZEN (Dall’Erta et al., 2013; Paris et al., 2014). More recently, several strains of *Bacillus* have been found capable of transforming ZEN to ZEN-14-phosphate (Zhu et al., 2021). Such a microbial transformation system may provide a new possibility for ZEN detoxification although the stability and toxicity reduction of ZEN-14-phosphate need to be further demonstrated in animal trials.

*Clonostachys rosea* (synonym: *Gliocladium roseum*) IFO 7063, was found to transform ZEN into a non-estrogenic product, 1-(3,5-dihydroxyphenyl)-10′-hydroxy-1′-undecen-6′-one (named DHZEN), through cleavage of the lactone ring followed by spontaneous decarboxylation (Figure 3C; Kakeya et al., 2002). The enzyme responsible for this activity, named ZHD101, was isolated and found to adopt an α/β-hydrolase fold with a typical catalytic triad of Ser-His and Asp found in many hydrolytic enzymes (Takahashi-Ando et al., 2002). Homologs of ZHD101 capable of hydrolyzing the lactone ring of ZEN were also found in other microbes, including *Cladophialophora bantiana* (Hui et al., 2017) and *Rhinocladiella mackenzieican* (Zheng et al., 2018). Another ZEN lactonase enzyme from *Rhodococcus erythropolis* was marketed as a feed additive by Biomin, Austria (Gruber-Dorninger et al., 2021). Recombinant *Saccharomyces cerevisiae*, *Escherichia coli* or *Lactobacillus reuteri* have been constructed that expressed these lactone hydrolases and found to hydrolyze ZEN to various extents (Takahashi-Ando et al., 2004; Yang et al., 2017; Liu et al., 2019). In particular, the genetically engineered *L. reuteri*, which expresses the lactonohydrolase, could potentially be used as a probiotic feed additive for the degradation of ZEN.

*Trichosporon mycotoxinivorans*, a basidiomycete yeast, was also found to cleave the lactone undecyl ring system at the ketone group at C7, leading to the formation of 5-({2,4-dihydroxy-6-[(1E)-5-hydroxypent-1-en-1-yl]benzoyl}oxy)hexanoic acid (named ZOM-1; Figure 3D; Vekiru et al., 2010). The transformation of ZEN to ZOM-1 was postulated to occur in two steps. First, a Baeyer-Villiger oxidation adds oxygen to the macrocyclic ring followed by hydrolysis of the resultant lactone. ZOM-1 was established to be non-estrogenic but the enzymes responsible for its formation from ZEN have not been identified.

## Citrinin

**Figure 4:**
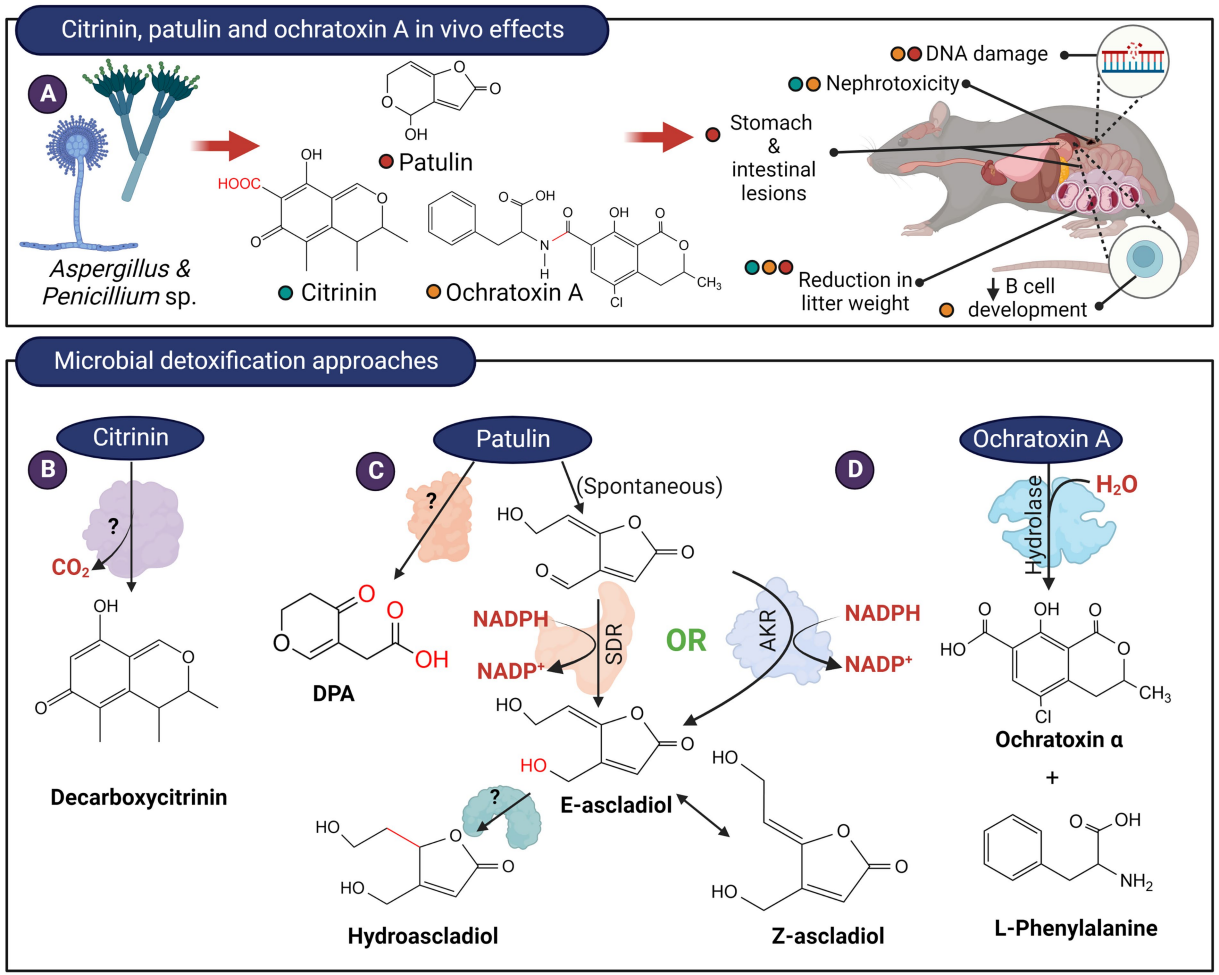
*In vivo* effects of citrinin, patulin, ochratoxin and microbial detoxification approaches. **(A)** These mycotoxins are produced by members of *Aspergillus* and *Penicillium* and typically, citrinin and ochratoxin possess nephrotoxic effects while patulin exerts serious gastrointestinal effects. **(B)** Decarboxylation of citrinin to decarboxycitrinin. **(C)** The hemiacetal ring of patulin likely exist in equilibrium with the ring-open form. The aldehyde in the ring-opened form of patulin can be reduced by enzymes from the short-chain dehydrogenase (SDR) or aldo-keto reductase (AKR) family to (E)-ascladiol. The E-ascladiol can be converted to the (Z)-isomer catalyzed by cellular sulfhydryl compounds, such as cysteine. Ascladiol can potentially be reduced to hydroascladiol by some microorganisms, such as *Lactobacillus plantanum.* Other basidiomycete yeasts can also transform patulin to DPA, although the formation of E-ascladiol appears to be more ubiquitous among various yeast and bacterial species. **(D)** The amide bond of ochratoxin A can be hydrolyzed to from L-phenylalanine and ochratoxin α.

Citrinin is a mycotoxin produced by fungi from the genus *Penicillium*, *Aspergillus,* and *Monascus* that mainly contaminate grains. The toxicity of citrinin is attributed mainly to its ability to induce oxidative stress (Vanacloig-Pedros et al., 2016) as well as its nephrotoxic properties (Shinohara et al., 1976; Figure 4A). A marine bacterial strain, *Moraxella* sp. MB1, is capable of transforming citrinin to decarboxycitrinin (Figure 4B; Devi et al., 2006). Decarboxycitrinin is not toxic when tested on mice (Jackson and Ciegler, 1978). The decarboxylating activity was observed in cell extract but not cell-free culture supernatant, suggesting that the unidentified enzyme responsible for this transformation is intracellular (Devi et al., 2006). Incidentally, decarboxycitrinin is a natural metabolite of the citrinin-producing fungus *Penicillium citrinum* and could also be produced by heat treatment of citrinin (Curtis et al., 1968; Jackson and Ciegler, 1978). Other microorganisms, such as *Rhizobium borbori* and *Klebsiella pneumoniae* NPUST-B11 have been reported to transform citrinin but the products have not been identified (Chen et al., 2011; Kanpiengjai et al., 2016).

## Patulin

Patulin is a bicyclic polyketide produced primarily by *Penicillium expansum* but can also be produced by other species of *Penicillium* as well as fungi belonging to the *Aspergillus* and *Byssochlamys* genus (Wright, 2015). The electrophilic α,β-unsaturated lactone present in patulin is reactive toward electron-rich sulfhydryl groups such as cysteine and glutathione, thereby affecting protein functions and increasing oxidative stress (Fliege and Metzler, 2000). The genotoxicity of patulin can also be attributed to the α,β-unsaturated lactone that induces double-stranded breaks in DNA strands (Pfenning et al., 2016). Short-term rat feeding studies also indicated that patulin directly affects the gastrointestinal tract as well as the kidneys (Speijers et al., 1988; Figure 4A). Patulin is largely found in apples and apple-based products but it can also contaminate other fruit products such as juices and jams as well as grain products and cheese (Pattono et al., 2013; Wright, 2015).

When incubated with patulin, certain microorganisms including *Saccharomyces cerevisiae* (Moss and Long, 2002), *Kodameae ohmeri* (Dong et al., 2015), *Gluconobacter oxydans* (Ricelli et al., 2007), and *Sporobolomyces* accumulate E-and/or Z-ascladiol (Ianiri et al., 2013). The reaction likely proceeds by the opening of the hemiacetal ring, followed by the reduction of the aldehyde group to alcohol (Figure 4C). It is thought that the Z-isomer of ascladiol is produced from E-ascladiol and was catalyzed by cellular sulfhydryl compounds such as glutathione and cysteine (Sekiguchi et al., 1983). An enzyme from the short-chain dehydrogenase (SDR) family has been isolated from *Candida guilliermondii* that catalyzed the transformation of patulin to E-ascladiol in an NADPH-dependent manner (Chen et al., 2017; Xing et al., 2021). The rate of patulin transformation by this enzyme appeared to be low as the enzyme at 150 μg/ml concentration took 72 h to fully transform 40 μg/ml of patulin. Enzymes related to the SDR family that can reduce patulin to E-ascladiol also appeared to be present in *Gluconobacter oxydans* (Ricelli et al., 2007; Lyagin and Efremenko, 2019). Chan et al., (2022) isolated 4 enzymes in *G. oxydans* that are capable of reducing patulin to E-ascladiol in an NADPH-dependent manner. Two of the enzymes (GOX0525 and GOX1899), belonging to the SDR family, was purified from recombinant *E. coli* and showed superior activity compared to the enzyme from *C. guilliermondii*. Recently, an aldo-keto reductase, originally isolated from *Devosia mutans* for the detoxification of the trichothecene, deoxynivalenol (DON; Carere et al., 2018b), was also found to be capable of reducing patulin to E-ascladiol (Abraham et al., 2022; Figure 4C). The catalytic efficiency of this enzyme, DepB, is 80-and 4-times lower compared to the SDRs GOX0525 and GOX1899 from *G. oxydans*. DepB can however utilize the less expensive NADH as a coenzyme, albeit less efficiently than NADPH.

Patulin can also be transformed to hydroascladiol (Figure 4C) by *Lactobacillus plantanum* (Hawar et al., 2013). Presumably, the ascladiol produced can be further reduced to hydroascladiol by certain microorganisms. Toxicity studies on hydroascladiol have not been reported.

In a transcriptomic study of *Pichia caribbica*, several genes, including *PcCRG1*, have been noted to be upregulated when incubated with patulin (Wang et al., 2019). *PcCRG1* codes for a methyltransferase that is dependent on the presence of S-adenosylmethione (S-Met) to transform patulin *in vitro* (Wang et al., 2019). Due to the necessity of S-Met, a methylated patulin was suggested to be the product of the reaction catalyzed by the enzyme (Wang et al., 2019). A knockout mutant of the *PcCRG1* gene had significantly reduced patulin transformation activity. Conversely, overexpression of the gene resulted in increased transformation activity (Wang et al., 2019). Interestingly, *P. caribbica* have been found to transform patulin into ascladiol *in vivo*, and not into a methylated patulin as expected from a PcCRG1 catalyzed reaction (Wang et al., 2019). This suggests that other enzymes are present in the fungus that can reduce patulin into ascladiol, but to date, an enzyme with this activity has not been isolated from the bacteria.

An orotate phophoribosyltransferase enzyme was isolated from *Rhodotorula mucilaginosa* and its patulin degrading activity was evaluated in apple juice (Tang et al., 2019). The degradation product from this enzyme has yet to be determined but it has been hypothesized to be a phosphoribosyl modified patulin, a metabolite of an unknown toxicity profile (Lyagin and Efremenko, 2019). However, an *in vivo* study on *R. mucilaginosa* has shown that ascladiol is the main byproduct of patulin degradation that suggests the activity of a reductase than a transferase (Yang et al., 2021).

Desoxypatulinic acid (DPA), is a less toxic metabolite of patulin that is produced by *Rhodosporidium kratochvilovae* (Castoria et al., 2011), *Rhodosporidium paludigenum* (Zhu et al., 2015), and *Sporobolomyces roseus* strain IAM 13481 (Ianiri et al., 2013). DPA appeared to be a metabolite of the hydrolyzed 5-membered lactone ring of patulin. The enzyme(s) responsible for transforming patulin to DPA has not been identified (Figure 4C).

## Ochratoxins

Ochratoxins, originally named from the producing strain *Aspergillus ochraceus*, are isocoumarin mycotoxin derivatives. They are also produced by species of *Aspergillus* and *Penicillium* and exist in three isoforms, ochratoxin A, B, and C. Of the 3 isoforms, ochratoxin A is the most potent, having nephrotoxic, immunosuppressive, teratogenic, and carcinogenic properties (Figure 4A).

The amide bond of ochratoxin A can be hydrolyzed to form phenylalanine and ochratoxin α, both of which are non-toxic (Figure 4D). The first enzyme discovered that can catalyze this reaction was bovine carboxypeptidase A, an enzyme with specificity toward peptides containing a terminal aromatic amino acid (Pitout, 1969). Subsequently, *Saccharomyces cerevisiae* carboxypeptidase Y was found to hydrolyze ochratoxin, albeit at a much lower rate compared to bovine carboxypeptidase A (Abrunhosa et al., 2010). Commercially available hydrolase preparations were then screened and one preparation from *Aspergillus niger*, marketed as Amano A lipase, was able to hydrolyze ochratoxin (Stander et al., 2000). Subsequent studies revealed that Amano A contained a mixture of enzymes, and the active enzyme against ochratoxin A is an amidase belonging to the amidohydrolase family of enzymes (Dobritzsch et al., 2014).

## Fumonisins

**Figure 5:**
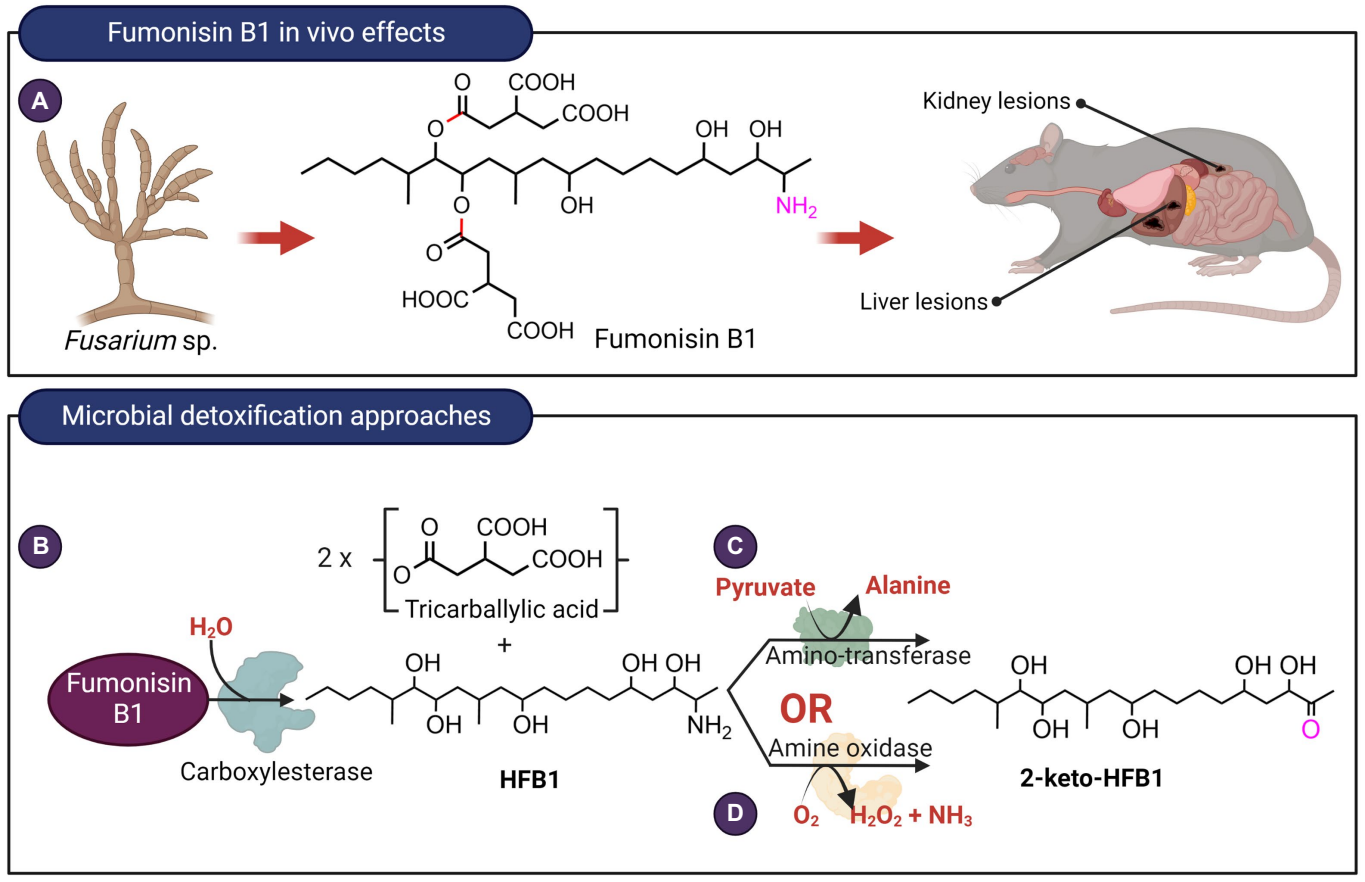
*In vivo* effects of fumonisin B1 and microbial detoxification approaches. **(A)** Fumonisin B1, typically produced by *Fusarium* species exert toxic effects on the liver as well as kidneys. **(B)** Hydrolysis of fumonisin B1 catalyzed by carboxyesterases target the ester linkages highlighted in red. Deamination reaction catalyzed by **(C)** aminotransferase or **(D)** an amine oxidase with the targeted C2 amino group highlighted in fuschia. The aminotransferase and amine oxidase from *Sphingopyxis* sp. MTA144 and *Exophiala spinifera* utilize hydrolyzed fumonisin B1 as substrate.

Fumonisins are aliphatic diesters that inhibit the enzyme ceramide synthase, thereby preventing sphingolipid biosynthesis. Currently, 28 fumonisin analogs have been identified differing in the substituents on the main chain (Alberts et al., 2016). Fumonisin B1 (FB1) is the most prevalent and toxic fumonisin. It is hepatoxic, nephrotoxic, and is a possible carcinogen (Figure 5A). The two tricarballylic acid moieties are essential for toxicity and they can be removed by hydrolysis of the ester bonds catalyzed by carboxylesterases present in bacteria and fungi. Of note is the carboxylesterase from the fumonisin-degrading bacterium *Sphingopyxis macrogoltabida* (Heinl et al., 2011) that has been commercialized as a feed additive by the company Biomin (Austria; Figure 5B). Although hydrolyzed FB1 (HFB1) is not toxic, the C2 amino group can be acylated by fatty acids *in vivo* to generate a ceramide synthase inhibitor that is 10-times more toxic than FB1 in mammalian HT-29 cells (Seiferlein et al., 2007). To prevent the acylation of the hydrolyzed FB1, the C2-amino group can be further deaminated. The bacterium, *Sphingopyxis* sp. MTA144, utilizes a pyridoxal phosphate-dependent amino transferase, with pyruvate as an amino acceptor to catalyze the deamination of HFB1 to produce 2-keto-HFB1 (Heinl et al., 2011; Figure 5C). The yeast, *Exophiala spinifera*, was also able to deaminate HFB1, and the enzyme that catalyzed the reaction was speculated to be an amine oxidase (Blackwell et al., 1999; Figure 5D). While amine oxidase requires oxygen for activity, it does not require a keto acid as a co-substrate, unlike aminotransferases.

Interestingly, certain fumonisin-producing *Aspergillus niger* accumulated deaminated fumonisin B1 in culture supernatant. The enzyme responsible for this reaction is later identified to be a monoamine oxidase and was found to also deaminate fumonisin derivatives, FB2 and FB3, with a preference for FB2 (Garnham et al., 2020). Although this enzyme-catalyzed a similar deamination reaction as the amine oxidase from *E. spinifera* discussed earlier, the two enzymes differed in two aspects. Firstly, *A. niger* monoamine oxidase deaminates fumonisin while the *E. spinifera* enzyme only deaminates HFB1. Secondly, *A niger* monoamine oxidase utilized FADH_2_ as a cofactor while amine oxidase typically requires topaquinone as a cofactor (Klinman, 2003).

## Trichothecenes

**Figure 6:**
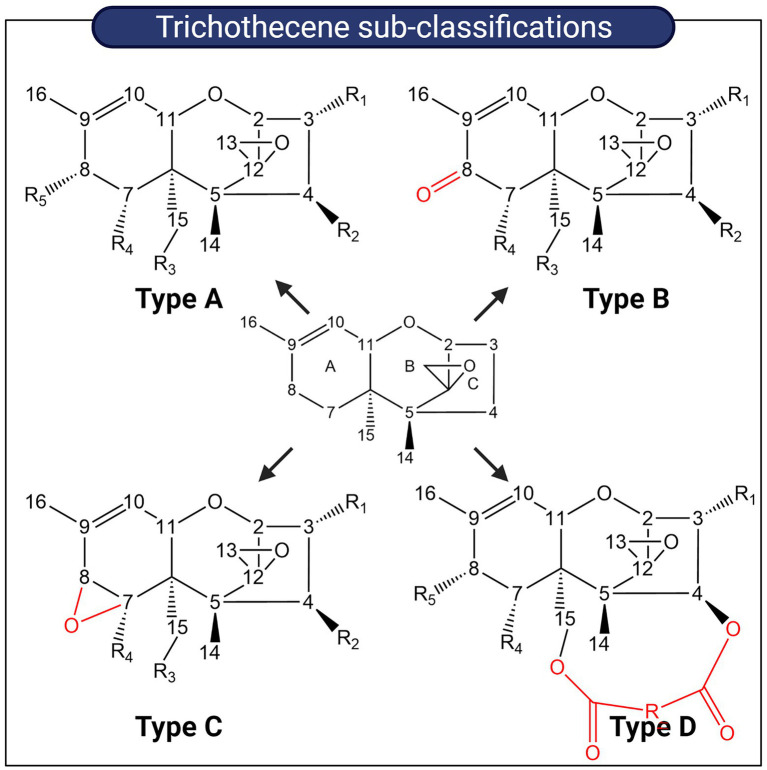
Sub-classification of trichothecenes. Trichothecenes can be sub-classified into four groups based on substitution patterns around the EPT nucleus. Key functional groups which distinguish each group from one another are highlighted in red.

Trichothecenes are tetracyclic sesquiterpenoid secondary metabolites primarily produced by members of the *Fusarium*, *Myrothecium*, *Stachybotrys*, and *Trichoderma* genera (Desjardins et al., 1993; Wilkins et al., 2003). The structural backbone is a fused tricyclic ring system comprising a cyclohexene ring (A-ring), a tetrahydropyran ring (B-ring), and a cyclopentyl ring (C-ring). An epoxide ring is attached to the tetrahydropyran ring and overall, this core structure is referred to as the 12,13-epoxytrichothec-9-ene (EPT) nucleus. Various substituent groups decorating the EPT nucleus are key to modulating the toxicity of trichothecenes and based on these substitution patterns, trichothecenes can be sub-classified into four groups designated Type A–D. Type A trichothecenes are distinguished from Type B which have a ketone group present on C8 of the EPT nucleus. Type C possesses an epoxide ring at C8 while Type D trichothecenes possess a di-or tri-ester linkage spanning the C4 and C15 regions (Shank et al., 2011; Figure 6).

**Figure 7:**
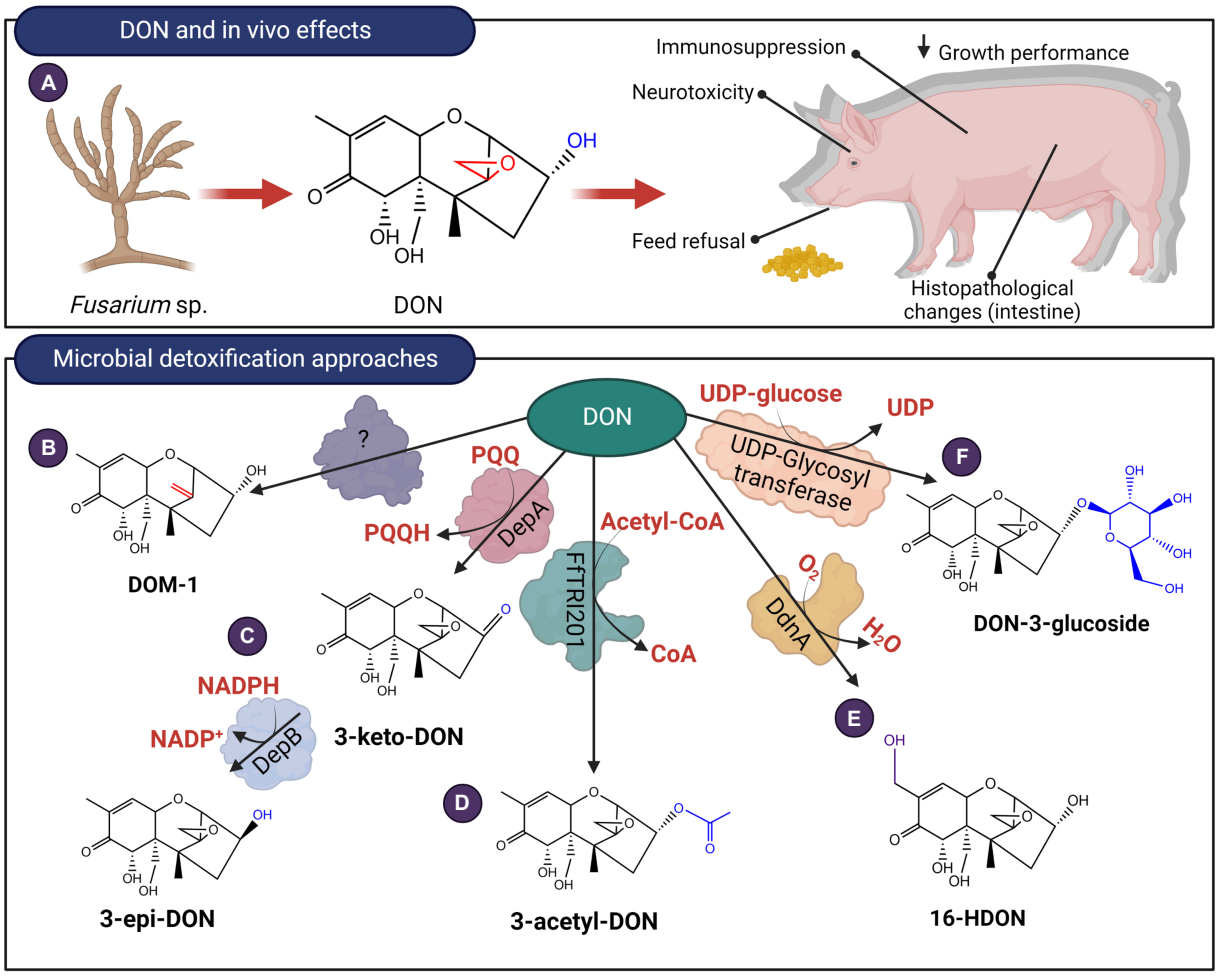
*In vivo* effects of Type B trichothecenes like DON and microbial detoxification approaches. **(A)** DON affects the gastrointestinal system and at a molecular level, the mycotoxin can bind to ribosomes triggering ribotoxic stress. **(B–F)** DON can be transformed by de-epoxidation, oxidation, epimerization, acetylation, hydroxylation and glycosylation.

**Figure 8:**
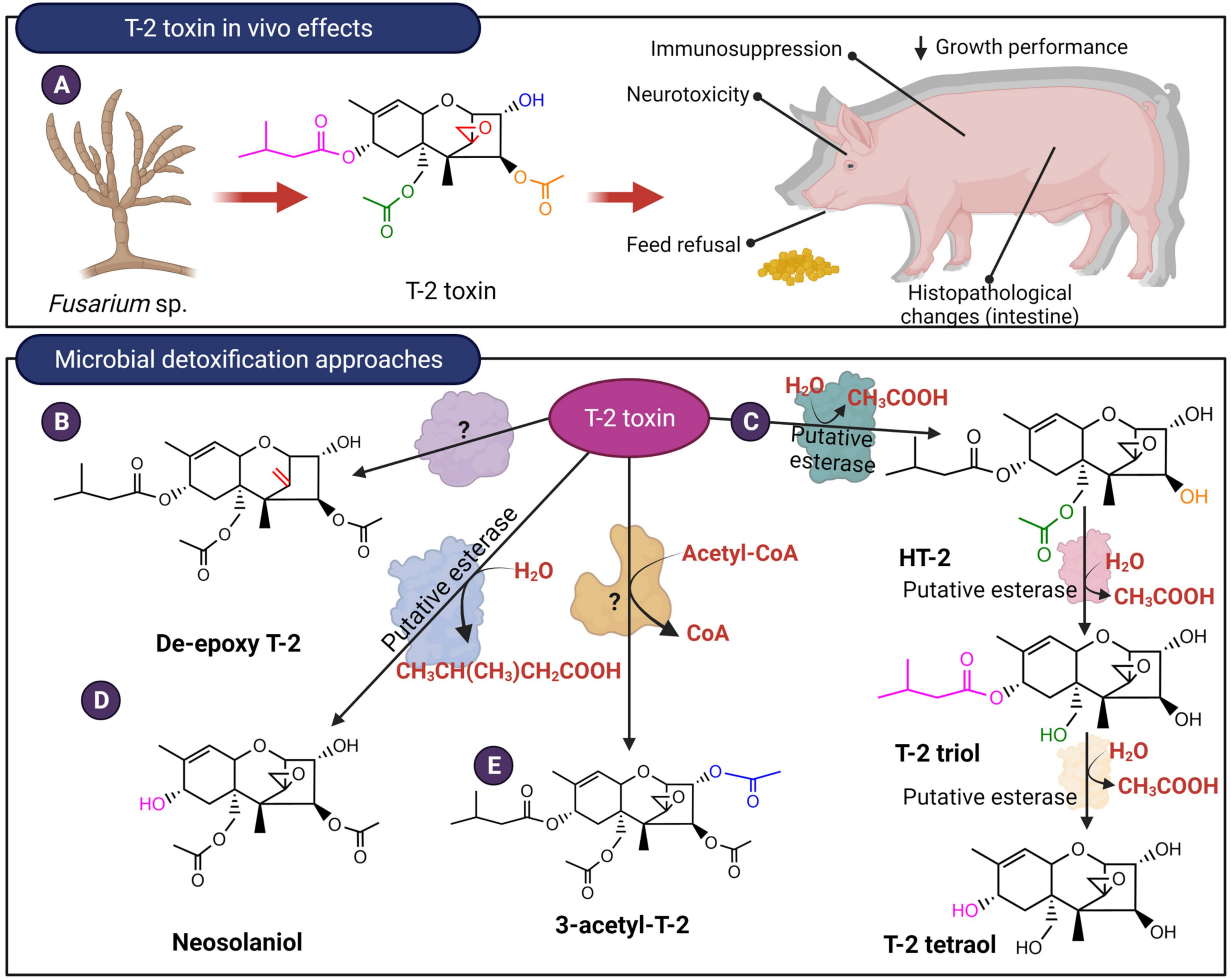
*In vivo* effects of Type A trichothecenes like T-2 toxin and microbial detoxification approaches. **(A)** T-2, like DON, causes negative gastrointestinal effects and ribotoxic stress. **(B–E)** The mycotoxin can be detoxified by de-epoxidation, hydrolysis of the ester linkages or acetylation.

Trichothecenes are small molecular inhibitors of protein synthesis and trigger ribotoxic stress by binding to the peptidyl transferase site of the large 60S subunit of eukaryotic ribosomes (Garreau De Loubresse et al., 2014). These interactions are mediated by the 12,13-epoxide ring, and for Type A trichothecenes such as T-2 toxin and Type B trichothecenes such as DON, the C3-hydroxyl is another contributing factor (Wang et al., 2021). Type A (T-2, HT-2, neosolaniol, diacetoxyscirpenol (DAS), monoacetoxyscirpenol (MAS), verrucarol (VER), scirpentriol (SCP)) and Type B trichothecenes (Deoxynivalenol (DON), nivalenol (NIV) and their acetylated derivatives) are the most prevalent chemotypes contaminating cereal grain crops (Foroud and Eudes, 2009). Ingestion of contaminated grain triggers gastrointestinal ailments in livestock and symptoms include diarrhea, emesis, feed refusal, and subsequent weight loss (Eriksen and Pettersson, 2004; Figures 7A, 8A). Limited studies exist for the detoxification of type C and D trichothecenes; instead, more research has been devoted to type A and B trichothecenes due to their significant agroeconomic impact. Microbial biotransformation of trichothecenes can occur through a variety of mechanisms including de-epoxidation, oxidation, epimerization, hydrolysis, acetylation, hydroxylation, and glycosylation.

De-epoxidation of trichothecenes has been primarily reported for anaerobic bacteria present in the gastrointestinal tract of ruminants and poultry or from soil environments. *Eubacterium* BBSH 797 commercialized as Biomin BBSH® 797 is a gram-positive, strict anaerobe isolated from ruminal fluid. This strain is proposed to conduct reductive de-epoxidation of the C12,13 epoxide ring of Type A and Type B trichothecenes such as T-2 and DON (Fuchs et al., 2000, 2002; Figures 7B, 8B). Since then, other microbial strains have been identified including *Bacillus* sp. LS100 (Yu et al., 2010), a consortium isolated from agricultural soil (Islam et al., 2012), a microbial consortium termed DX100 (Ahad et al., 2017), *Eggerthella* sp. DII-9 (Gao et al., 2018), *Desulfitobacterium* sp. strain PGC-3-9 (He et al., 2020), and *Slackia* sp. D-G6 (Gao et al., 2020). The reduced toxicity of de-epoxy derivatives of T-2 and DAS was demonstrated using a brine shrimp toxicity bioassay that showed higher LC_50_ values (Swanson et al., 1987). Likewise, cytotoxicity assays of de-epoxy derivatives of DON (named DOM-1) and NIV showed IC_50_ values that were 52 and 51 times higher than the parent mycotoxins, respectively (Eriksen and Pettersson, 2004). To date, the isolation of enzymes responsible for the reductive de-epoxidation of trichothecenes has remained elusive. Comparative genomics of *Slackia* sp. with closely related strains that showed no DON de-epoxidation identified 13 possible gene clusters that may be responsible for de-epoxidase activity (Gao et al., 2020). Five of these clusters were recombinantly expressed but no *in vitro* trichothecene de-epoxidation activity was observed. The recombinant strain used was not indicated and it is unclear if the lack of activity is due to poor heterologous expression of the genes.

The C3 OH of DON can be epimerized from the R configuration to the S configuration (Hassan et al., 2017). The resulting diastereomer, 3-*epi*-DON, interacts less strongly with ribosomes with an IC_50_ 357 times higher than DON (He et al., 2015; Wang et al., 2021). *In vivo* studies were also conducted for pigs fed diets spiked with either DON, de-epoxy DON (DOM-1), or 3-*epi*-DON. Treatments containing either DOM-1 or 3-*epi*-DON did not show significant morphological changes in the intestine, nor did they elicit a strong immunological response when compared with DON (Bracarense et al., 2020). Certain strains belonging to *Nocardiodes* (Ikunaga et al., 2011) *Sphingomonas* (He et al., 2017), and *Devosia* (He et al., 2015; Hassan et al., 2017) were shown to aerobically detoxify DON in this manner. In *Devosia mutans* 17-2-E-8, the enzymes responsible for epimerization were identified and recombinantly expressed in *E. coli*. Oxidation is first conducted by DepA (for DON Epimerization), a Type I pyrroloquinoline quinone-dependent alcohol dehydrogenase (PQQ-ADH; Carere et al., 2018a). The subsequent step involves DepB, an aldo-keto reductase that stereo-specifically reduces 3-keto-DON to 3-*epi*-DON in an NADPH-dependent manner (Carere et al., 2018b; Figure 7C). Homologs of DepB have been identified in *Sphingomonas* sp. S3-4 (He et al., 2017), *Devosia* sp. strain D6-9 (He et al., 2020) and *Rhizobium leguminosarum* (Abraham et al., 2022). Unlike DepB, its homolog from *Sphingomonas* sp. S3-4 oxidizes DON, while a second, unidentified enzyme is believed to stereo-specifically reduce 3-keto-DON to 3-*epi*-DON, however, this enzyme has not been isolated to date. While epimerization remains a promising approach for enzymatic detoxification of trichothecenes such as DON, its feasibility is limited due to the requirement for cofactors PQQ and NADPH. Interestingly, DepB_Rleg_ can reduce 3-keto-DON to 3-*epi*-DON with NADH, albeit with a catalytic efficiency 40-fold lower than with NADPH (Abraham et al., 2022).

Certain bacteria can oxidize DON to 3-keto-DON but were not capable of transforming 3-keto-DON to 3-*epi*-DON. These bacteria include strain E3-39 from the *Agrobacterium-Rhizobium* group of bacteria (Shima et al., 1997), a mixed culture termed D107 (Völkl et al., 2004) and *Pelagibacterium halotolerans* ANSP101 (Zhang et al., 2020). The enzymes responsible for transforming DON to 3-keto-DON in these bacteria have not been identified. MTT bioassays showed that 3-keto-DON has an IC_50_ value of at least 3 times higher compared with DON but is still significantly more toxic than 3-*epi*-DON (He et al., 2015).

*Curtobacterium* sp. strain 114–2 was reported to utilize T-2 toxin as a carbon and energy source (Ueno et al., 1983). The strain hydrolyzed the ester linkage at the C4 position to produce HT-2 toxin which is in turn hydrolyzed at the C15 ester linkage to produce T-2 triol. This intermediate is finally degraded to unknown metabolic products (Figure 8C). *In vivo* toxicology studies showed that T-2 triol possesses an LD_50_ 23 and 13 times higher in comparison with T-2 and HT-2 toxins, respectively (Ueno et al., 1983). This hydrolysis was attributed to putative esterases and activity was confirmed for both culture supernatants as well as whole cells, however, the extracellular enzymes proved to be unstable. *Curtobacterium* sp. strain 114-2 was also capable of transforming DAS to produce scirpentriol by targeting the ester linkages at the C4 and the C15 positions. In contrast, in the ruminal anaerobe *Butyrivibrio fibrisolvens*, only the C4 ester linkage can be hydrolyzed producing 15-acetoxyscirpenol (Matsushima et al., 1996). Other microbial consortiums from soil and freshwater can further hydrolyze T-2 triol to produce T-2 tetraol (Figure 8C), as well as transform T-2 into neosolaniol (Beeton and Bull, 1989). In terms of toxicity, T-2 tetraol is about 20-fold less toxic than T-2 toxin when administered to mice (Yoshizawa et al., 1984). Similarly, yeast species from the *Trichomonascus* clade, namely *Blastobotrys capitulata, Blastobotrys mokoenaii, and Blastobotrys malaysiensis,* converted T-2 toxin into neosolaniol by removal of the C8 isovaleryl group (Figure 8D). Certain species within this clade were also able to modify the C3 OH of T-2 toxin and neosolaniol to form an acetylated derivative (McCormick et al., 2012; Figure 8E). To date, specific enzymes that hydrolyze the ester linkages or modify the C3 OH of trichothecenes have not been isolated from these yeast species.

Acetylated precursors of trichothecenes are formed during the final stages of their synthesis as a self-protection mechanism for the trichothecene-producing fungi (Kimura et al., 1998). Acetylation of DON to mitigate levels in distillers dried grains with solubles (DDGS) has been demonstrated by recombinantly expressing these trichothecene 3-O-acetyltransferases in *Saccharomyces cerevisiae* strain RW2802 (Khatibi et al., 2011). Among the acetyltransferases examined, FfTRI201, an ortholog of TRI101 from *F. graminearum* showed the highest conversion of DON to 3-acetyl-DON (3-ADON; Figure 7D). The 3-O-acetylated derivatives showed 100-fold higher IC_50_ values relative to the parent mycotoxins when administered to rabbit reticulocytes (Kimura et al., 1998).

Hydroxylation of DON at the C16 methyl group had been demonstrated for a *Sphingomonas* strain KSM1 isolated from a lake in Japan (Ito et al., 2013; Figure 7E). The hydroxylation reaction was determined to involve a cytochrome P450 oxidase (ddnA), an NADH-dependent ferredoxin reductase KdR and ferredoxin Kdx (Ito et al., 2013). When all three proteins were heterologously expressed in *E.coli* Rosetta 2 (DE3), they oxidized DON to 3α,7α,15,16-tetrahydroxy-12,13-epoxytrichothec-9-en-8-one or 16-HDON (Ito et al., 2013). Phytotoxicity assays showed that 16-HDON had a 10-fold reduced toxicity in comparison to DON.

DON glycosylation at the C3 OH position is a phase II detoxification mechanism typically employed by plants. The enzyme, a UDP-glucosyltransferase, transfers glucose from the substrate, UDP-glucose to an alcohol group, producing DON-3-glucoside (D3G; Poppenberger et al., 2003; Figure 7F). Recent evidence suggests that fungi such as *Trichoderma* sp. (Tian et al., 2016) and *Clonostachys rosea* (Demissie et al., 2020) also adopt a similar strategy to detoxify DON as well as its acetylated derivative, 15-acetyl-deoxynivalenol (15-ADON). Plate confrontation assays with either *Trichoderma* strains or *C. rosea* against *F. graminearum*, resulted in the accumulation of D3G and 15-acetyl-DON-3-glucoside, respectively. It is postulated that either an extracellular protein is involved, or a glycosyl-transferase, which affixes a glucose moiety to DON or 15-ADON. These metabolites are subsequently exported to the media *via* transporter proteins. The growth rates of *S. cerevisiae* and *Chlamydomonas reinhardtii* supplemented with D3G revealed no significant effects, demonstrating the attenuated toxicity of the glycosylated derivative (Suzuki and Iwahashi, 2015).

## Practical aspects and future prospects

A wide variety of microbial enzymes are capable of transforming mycotoxins to less toxic compounds (Table 2). Mycotoxins, such as ZEN, contain hydroxyl substituents that can be conjugated *via* ester linkages to sugars or sulfate (Kamimura, 1986; Plasencia and Mirocha, 1991; Paris et al., 2014). However, conjugated mycotoxins are labile and could be hydrolyzed to the parent mycotoxins in the gut by acid/alkaline or intestinal microbiota. Therefore, enzymes that form mycotoxin conjugates are generally not reliable as detoxification strategies.

**Table 2:** Summary of isolated and putative enzymes involved in mycotoxin detoxification.

Mycotoxin	Enzyme (s)	Enzyme requirement	Mechanism	Microorganism	References
Aflatoxin B1	Laccase manganese peroxidase	Redox mediators, H_2_O_2_ or O_2_	Mycotoxin degradation may occur through free radical generation	*Pleurotus eryngii, Pleurotus pulmonarius, Bacillus licheniformis* ANSB821, *Phanerochaete sordida* YK-624, *Rhodococcus jostii*	Loi et al. (2016), (2018), Guo et al. (2020), Wang et al. (2011), and Vignali et al. (2018)
	F_420_H_2_-dependent reductases (FDR)	Reducing agents for regeneration of F_420_H_2_ cofactor	Double bond reduction of α,β unsaturated ring A	*Mycobacterium smegmatis*	Taylor et al. (2010)
Zearalenone	Unknown	Unknown	Phosphorylation	*Bacillus* sp.	Zhu et al. (2021)
	Lactonase	None	Lactone ring hydrolysis	*Clonostachys rosea* IFO 7063*, Cladophialophora bantiana, Rhinocladiella mackenzieican, Rhodococcus erythropolis*	Kakeya et al. (2002), Hui et al. (2017), Zheng et al. (2018), and Gruber-Dorninger et al. (2021)
	Two putative enzymes: 1. Baeyer-Villiger monooxygenase 2. Lactonase	NADPH and oxygen required for the BVMO	Baeyer-Villiger oxidation followed by lactone hydrolysis	*Trichosporon mycotoxinivorans*	Vekiru et al. (2010)
Citrinin	Unknown	Unknown	Decarboxylation	*Moraxella* sp. MB1	Devi et al. (2006)
Patulin	Short-chain dehydrogenase Aldo-keto reductase	NADPH	Reduction of the product following hemiacetal ring opening	*Candida guilliermondii Gluconobacter oxydans* ATCC 621 *Rhizobium leguminosarum*	Chen et al. (2017), Xing et al. (2021), Chan et al. (2022), and Abraham et al. (2022)
Ochratoxin	Carboxy-peptidase Y	None	Hydrolysis of the amide bond	*Saccharomyces cerevisiae*	Abrunhosa et al. (2010)
	Amido-hydrolase			*Aspergillus niger*	Stander et al. (2000) and Dobritzsch et al. (2014)
Fumonisins	Carboxyl-esterase	None	Hydrolysis of ester bond linkage to tricarballylic acid	*Sphingopyxis macrogoltabida*	Heinl et al. (2011)
	Amino-transferase	Pyridoxal phosphate and pyruvate	Deamination	*Sphingopyxis* sp. MTA144	Heinl et al. (2011)
	Amine oxidase	Oxygen		*Exophiala spinifera*	Blackwell et al. (1999)
	Monoamine oxidase	Reducing agent for regeneration of FADH_2_		*Aspergillus niger*	Garnham et al. (2020)
DON	Unknown	Unknown	Reductive de-epoxidation	*Eubacterium* BBSH 797, *Bacillus* sp. LS100, mixed culture from soil, Consortium DX100, *Desulfitobacterium* sp. PGC-3-9, *Slackia* sp. D-G6, *Eggerthella* sp.DII-9	Fuchs et al. (2000), Fuchs et al. (2002) Yu et al. (2010), Islam et al. (2012), Ahad et al. (2017), He et al. (2020), Gao et al. (2020), and Gao et al. (2018)
	1. Type I PQQ dependent alcohol dehydrogenase, DepA (including recently identified homologs) 2. Aldo-keto reductase, DepB (including recently identified homologs)	1. PQQ 2. NADPH	Epimerization	*Devosia mutans* 17-2-E-8, *Devosia* sp. strain D6-9, *Rhizobium leguminosarum*	Carere et al. (2018a,b), He et al. (2020), and Abraham et al. (2022)
	AKR18A1, second enzyme not identified	NADP^+^	Oxidation	*Sphingomonas* sp. strain S3-4	He et al. (2017)
	Acetyl-transferase, FfTRI201	Acetyl-CoA	Nucleophillic substitution	*Fusarium fujikuroi* IFO 31251	Khatibi et al. (2011)
	Three enzymes: 1. DdnA 2. KdR 3. Kdx	NADH	Hydroxylation	*Sphingomonas* sp. strain KSM1	Ito et al. (2013)
	Unknown	Unknown	Glycosylation	*Trichoderma* sp. *Clonostachys rosea*	Tian et al. (2016), Demissie et al. (2020)
T-2 toxin	Unknown	Unknown	Reductive de-epoxidation	*Eubacterium* BBSH 797	Fuchs et al. (2000) and Fuchs et al. (2002)
				Consortium DX100	Ahad et al. (2017)
	Putative esterase(s)	None	Hydrolysis	*Curtobacterium* sp. strain 114–2	Ueno et al. (1983)

Other enzymes such as oxidases, reductases, and amino transferases require cofactors or co-substrates, such as NADH or hydrogen peroxide, for activity. This adds additional costs, introduces chemical residues, and complicates the scale-up of the mycotoxins detoxification process. In the biotechnology industry, a secondary enzyme such as formate dehydrogenase has been explored for regenerating NADH from NAD^+^ that will provide a reduced cofactor for reductase/dehydrogenase reactions (Hummel and Gröger, 2014). Formate will simultaneously be converted to carbon dioxide gas that is easily removed in the reaction. The application of this coenzyme recycling strategy for the enzymatic detoxification of mycotoxins is worth exploring. Alternatively, the use of whole microorganisms instead of purified enzymes could circumvent the need to supplement coenzymes or co-substrates for biotransformation as they can be generated *in situ* within the microbial cells. Furthermore, increasing the intracellular pool of coenzymes such as NADPH may be achieved through metabolic engineering approaches as described in other reviews (Lee et al., 2013).

However, precautions must be taken to ensure that these microorganisms are not pathogenic and do not have secondary activities that significantly alter the composition of the food product. Novel microbes destined for use in the food industry may modulate the gut microbiota, hence, these candidates must be screened for virulence, antibiotic resistance profiles as well as their propensity for the production of antimicrobial compounds (Brodmann et al., 2017).

The use of whole microorganisms for mycotoxin detoxification is particularly beneficial if these microorganisms are already in use in the food production process, such as in fermentation. For example, *Saccharomyces cerevisiae* has been demonstrated to degrade patulin during fermentation to ascladiol (Moss and Long, 2002). As a result, the final processed product of cheeses and alcoholic ciders was observed to not experience significant patulin contamination, although the raw materials were initially contaminated with the mycotoxin.

Several mycotoxins contain lactone rings, amide, or ester linkages that can be hydrolyzed by specific enzymes. The hydrolytic reaction requires an aqueous environment but no co-substrates or coenzymes are necessary. The simplicity of the hydrolytic reaction likely contributed to the successful commercialization of purified hydrolases against fumonisin and ZEN applied to feed additives (Heinl et al., 2011; Gruber-Dorninger et al., 2021).

Advances in mass spectrometry techniques to interrogate the proteome coupled with genome sequencing and analysis should continue to accelerate the discovery of microbial enzymes with mycotoxin transformation activity in the future (Chen et al., 2017; Sandlin et al., 2022). This will provide a robust selection of enzymes that could be used for mycotoxin detoxification.

## Author contributions

NA and SS were involved in the development of the topic and initial drafts. EC and TZ edited and added additional information to the draft. TZ conceived and coordinated the study. All authors contributed to the article and approved the submitted version.

## Funding

This study was supported by Agriculture and Agri-Food Canada (AAFC J-002250) and Grain Farmers of Ontario through the Industry-led Research and Development Project (J-002433).

## Conflict of interest

The authors declare that the research was conducted in the absence of any commercial or financial relationships that could be construed as a potential conflict of interest.

## Publisher’s note

All claims expressed in this article are solely those of the authors and do not necessarily represent those of their affiliated organizations, or those of the publisher, the editors and the reviewers. Any product that may be evaluated in this article, or claim that may be made by its manufacturer, is not guaranteed or endorsed by the publisher.
